# *Rhizobium etli* CFN42 and *Sinorhizobium meliloti* 1021 bioinformatic transcriptional regulatory networks from culture and symbiosis

**DOI:** 10.3389/fbinf.2024.1419274

**Published:** 2024-08-28

**Authors:** Hermenegildo Taboada-Castro, Alfredo José Hernández-Álvarez, Juan Miguel Escorcia-Rodríguez, Julio Augusto Freyre-González, Edgardo Galán-Vásquez, Sergio Encarnación-Guevara

**Affiliations:** ^1^ Center for Genomic Sciences, National Autonomous University of México, Cuernavaca, Mexico; ^2^ Institute of Applied Mathematics and in Systems (IIMAS), National Autonomous University of México, Mexico City, Mexico

**Keywords:** transcriptional, regulatory, network, motif, symbiosis, rhizobia, *etli*, nitrogen-fixation

## Abstract

*Rhizobium etli* CFN42 proteome–transcriptome mixed data of exponential growth and nitrogen-fixing bacteroids, as well as *Sinorhizobium meliloti* 1021 transcriptome data of growth and nitrogen-fixing bacteroids, were integrated into transcriptional regulatory networks (TRNs). The one-step construction network consisted of a matrix-clustering analysis of matrices of the gene profile and all matrices of the transcription factors (TFs) of their genome. The networks were constructed with the prediction of regulatory network application of the RhizoBindingSites database (http://rhizobindingsites.ccg.unam.mx/). The deduced free-living *Rhizobium etli* network contained 1,146 genes, including 380 TFs and 12 sigma factors. In addition, the bacteroid *R. etli* CFN42 network contained 884 genes, where 364 were TFs, and 12 were sigma factors, whereas the deduced free-living *Sinorhizobium meliloti* 1021 network contained 643 genes, where 259 were TFs and seven were sigma factors, and the bacteroid *Sinorhizobium meliloti* 1021 network contained 357 genes, where 210 were TFs and six were sigma factors. The similarity of these deduced condition-dependent networks and the biological *E. coli* and *B. subtilis* independent condition networks segregates from the random Erdös–Rényi networks. Deduced networks showed a low average clustering coefficient. They were not scale-free, showing a gradually diminishing hierarchy of TFs in contrast to the hierarchy role of the sigma factor *rpoD* in the *E. coli* K12 network. For rhizobia networks, partitioning the genome in the chromosome, chromids, and plasmids, where essential genes are distributed, and the symbiotic ability that is mostly coded in plasmids, may alter the structure of these deduced condition-dependent networks. It provides potential TF gen–target relationship data for constructing regulons, which are the basic units of a TRN.

## Introduction

*Rhizobium etli* CFN42, a free-living soil bacterium, is an alphaproteobacterium able to establish a symbiotic relationship with leguminous plants (Andrews and Andrews, 2017; Lardi and Pessi, 2018; Dicenzo et al., 2019). *R. etli* CFN42 lives in a wide variety of environmental conditions; in the rhizosphere, the bacterial community competes for nutrients from the soil and exudates of the tropical plant *Phaseolus vulgaris*; this may be a limited nutrient condition. In symbiosis, a completely different living condition is experienced for rhizobia, which commences with a chemical communication and signaling between rhizobia and the plant; rhizobia is allocated inside the plant cells of nodules, and the nodules are elicited by the bacterium on the root from the leguminous plant, where rhizobia is differentiated to a bacteroid giving rise to the organelle symbiosome (Mylona et al., 1995; Van Rhijn and Vanderleyden, 1995; Rascio and La Rocca, 2013; Dicenzo et al., 2019). This pleomorphic bacteroid converts the atmospheric di-nitrogen (N_2_) to ammonium, called symbiotic nitrogen fixation (SNF); the ammonium is exported to the plant cells in an exchange of carbon compounds supplied by the plant cells from photosynthesis, and the bacteroid metabolizes this photosynthate to sustain the nitrogen fixation (Rascio and La Rocca, 2013). The symbiont *Rhizobium*, as an inoculant of leguminous plants, is capable of substituting the use of chemical fertilizers for the production of grains for human consumption and pastures for animal breeding, which is a cheaper alternative to industrial nitrogen fertilizers that pollute the environment, having a significant impact on ecosystems and in the community (Oldroyd et al., 2011; Ferguson et al., 2019).

Efficient survival of rhizobia in different living conditions involves coordinated metabolic responses through transcriptional regulation of genes and appropriate protein quantity of proteins (Barabási and Oltvai, 2004; Escorcia-Rodríguez et al., 2020). A TRN comprises different components, including global transcriptional regulators, modules containing regulons and transcriptional regulators (TFs), genes involved in metabolic processes, basal machinery genes, and intermodular genes. These modules respond to environmental cues and integrate their responses at the promoter level (Freyre-González et al., 2012; Ibarra-Arellano et al., 2016; Escorcia-Rodríguez et al., 2021; Freyre-González et al., 2022).

TFs bind to a short, specific nucleotide sequence called a motif located in the promoter sequence of the target genes; then, identifying the motifs becomes a crucial task for a TRN. The ChIP-seq experimental method allows the identification of motifs at the genomic level. However, it is an expensive technique, and once the motif is determined, the next challenging step is to identify the TF (Cuesta-Astroz et al., 2021). Alternatively, bioinformatic methods become relevant to address these questions at the genomic level, for example, identifying the SNF regulons.

To construct a TRN of the SNF, knowledge of transcriptional regulation at the genomic level is needed for genetic modifications in symbiotic species to enhance nitrogen fixation of rhizobia distributed in a wide variety of geographic conditions.

Great progress was made on the O_2_-dependent regulation of the SNF by extending known motifs with bioinformatic methods to establish the *NifA-RpoN* regulon of nitrogen fixation in the alphaproteobacteria group, and the deduced matrices from the motifs of the TFs inferred with a highly strict *p*-value were used in RegPredict site to define the operons (Novichkov et al., 2010). This search yielded 95 operons that potentially contain NifA-binding sites, including 280 genes (Tsoy et al., 2016). In addition, the NifA-RpoN regulon of *R. etli* CFN42 was determined by experimental and bioinformatic methods, and it was found to consist of 78 genes (Salazar et al., 2010). Comparing the number of genes from both studies, it appears that the study of the NifA regulon in alphaproteobacteria is highly conservative. This suggests that considerable data regarding the *in silico* deduced regulon may have been eliminated.

A computational analysis based on protein–protein interactions of *Sinorhizobium meliloti* with its host plants proposed the symbiosis “interactome,” which was composed of 440 proteins involved in 1,041 unique interactions (Rodriguez-Llorente et al., 2009).

In addition, proteomic studies on symbiosis have been reported (Larrainzar and Wienkoop, 2017; Lardi and Pessi, 2018; Khatabi et al., 2019). Although it contains protein profiles of symbiotic and free-living organisms, including transcription factors (TFs), it is necessary to know the TF gene–targets to create genetic circuitry with these profiles.

TFs gene–targets with computationally predicted motif were used to construct TRNs of *R. etli* CFN42 protein profiles after 6 h of growth in a minimal medium and 18 days post-inoculation. A clustered-TF network was constructed using a method that included an initial network followed by matrix-clustering analysis and clustered-TF gene–targets. However, in the *R. etli* CFN42 clustered-TF gene–targets for the second network, there was a significant reduction of genes compared to the first network, probably because a significant number of TFs involved in regulating the genes of the profile were absent. Low modularity was detected for these networks, probably affected by a low number of genes and a low number of inter-modular genes, particularly in the bacteroid profile (Taboada-Castro et al., 2022).

In this study, 1.7 and 3.59 more genes were included to address the diminishing of genes. The method to construct a network consisted of a matrix-scan analysis of the matrices of the gene profile and matrices of all TFs of the corresponding genome. Properties of the deduced *R. etli* CFN42 and *S. meliloti* 1021 networks, together with the experimentally constructed *Escherichia coli*, *Bacillus subtilis*, and their corresponding Erdös–Rényi random networks, were contrasted (Escorcia-Rodríguez et al., 2020).

## Material and methods

### Design of the regulatory network

In contrast to our last method for constructing the TRN, which consisted of three steps (Taboada-Castro et al., 2022), in this report, a one-step method was assayed, which consisted of an analysis of matrices with the matrix-clustering method of the genes from each profile together with all matrices of TFs available of the respective genome to avoid the drastic diminishing of genes during the construction of the network (see above). Matrices of the significative genes from the protein and transcriptome profiles of *R. etli* CFN42 grown in minimal medium (MM) at 6 h and of bacteroid isolated from nodules at 18 days post-inoculation of the bean plant *Phaseolus vulgaris* (matrices available under request) as well as the matrices of all TFs of the respective genome were taken (matrices from TFs are available in the section “matrix-clustering” of the respective species) (Salazar et al., 2010; Taboada-Castro et al., 2022). These data were the input for the matrix-clustering program available on the RSAT web page (http://embnet.ccg.unam.mx/rsat/matrix-clustering_form.cgi). This program constructs clusters based on similarity in hierarchical dendrograms using the HCLUST algorithm (Castro-Mondragon et al., 2017). There is an output file “clusters_motif_names.tab” containing the clusters in a list (i.e., cluster_3 RHE_RS06555_m1, RHE_RS06555_m2, RHE_RS03090_m3, RHE_RS03090_m1, and RHE_RS03090_m2). Notably, genes RHE_RS06555 and RHE_RS03090 appeared repeatedly in this cluster (data not shown). To avoid this redundancy of genes, unique genes per cluster were extracted from the clusters_motif_names.tab output file, and clusters with at least two different genes were extracted; subsequently, the TF or TFs were identified per cluster (Supplementary Tables 1, 2, a and b). In this step, all the genes with a similar motif shared with a motif of a TF were retained, augmenting the possibilities of predicting TF gene–targets with motif consensus conserved from their respective ortholog genes and eliminating probably false-positive data. For the construction of a network in the application “Prediction of regulatory networks” from the RhizoBindingSites database located in the windows “motif information” of the corresponding species, that is, for *R. etli* CFN42 networks (http://rhizobindingsites.ccg.unam.mx/Motif.jsp?v=v1&s=1), all unique TFs from the matrix-clustering were copy-pasted in the left box, and a mix of all unique targets and unique TFs in the right box of the application, for example, for the *R. etli* CFN42 MM network (see Supplementary Table 1, columns E and I), the “auto” option was selected and run. The program looks for all the targets in the 1e-06 to lower *p*-value data with each TF (highest stringency data). All the genes that were targeted by a TF or TFs were separated from the list, and the rest of the genes were the input for a new search in the 1e–05 *p*-value data with all the TFs (medium stringency data), and again, all targeted genes were separated from the list. Finally, the remaining genes were the input for a new search for targets in the 1e–04 *p*-value data (low-stringency data). These networks were constructed with the lowest *p*-value possible TF gene–target relationships and, consequently, with the highest stringency, called “clustered-TF networks” (Supplementary Tables 3–6, a).

For the construction of *Sinorhizobium meliloti* 1021 clustered-TF free-living and bacteroid networks, an identical method was applied to the matrices of significative genes from the transcriptome data of growth in a complete tryptone yeast extract medium between 0.5 and 0.7 OD600 and bacteroid detached from the roots of the alfalfa host plant *Medicago truncatula* at 33–35 days of symbiosis from *S. meliloti* 1021, respectively (Supplementary Tables 5, 6) (Barnett et al., 2004).

### Gene co-expression network

The gene co-expression network was built using the WGCNA algorithm using a collection of 40 gene expression samples from the Collections of Microarrays for Bacterial Organisms (COLOMBOS) (Langfelder and Horvath, 2008; Moretto et al., 2016). The scale-free topological features of biological networks were detected using pickSoftThreshold with a power (β) of nine.

Subsequently, an adjacency matrix was created by utilizing the signal correlation matrix and the pairwise biweight midcorrelation coefficients between each gene, where nodes with negative correlations are regarded as disconnected. This correlation approach was selected as it outperforms the Spearman and Pearson correlation methods (Bakhtiarizadeh et al., 2018). Next, a topological overlap matrix (TOM) was created using the adjacency matrix. A higher TOM value made it possible to identify the gene modules for each pair of strongly interconnected genes.

Finally, the average linkage hierarchical clustering technique (flashClust function) was used to group the genes into modules with similar expression patterns. The branches of the resultant dendrogram, which produces the gene modules, were then cut using the cutreeDynamic function. The distance matrix 1-TOM, whose minimum module size was 20, was employed to achieve this. Consequently, the modules exhibiting a strong correlation between their eigengenes were combined using the mergeCloseModules function, with a minimum height of 0.25. Each module was assigned a color and uncorrelated genes were assigned gray (Horvath, 2011).

### Properties of networks

We computed several global structural properties for regulatory networks, namely, regulators ( $k_{out}>0$ ), self-regulations, maximum out-connectivity, giant component size, network density, feedforward circuits, complex feedforward circuits, 3-feedback loops, average shortest path length, network diameter, average clustering coefficient, adjusted coefficient of determination ( $R_{adj}^{2}$ ) of $P\left(k\right)$ , and $R_{adj}^{2}$ of $C\left(k\right)$ . Regulators, self-regulations, maximum out-connectivity, and giant component size were normalized by the number of nodes in the network. The density was included as the product of the network density and the fraction of regulators. Network diameter was normalized (number of nodes, 2) (as if no shortcuts would exist). The 3-feedback loops, feedforward loops, and complex feedforward loops were normalized by the number of potential motifs in the network, defined as

$$\frac{n!}{\left(n-r\right)!}\cdot {\left(\frac{{TF}_{n}}{n}\right)}^{{TF}_{m}},$$

where n is the number of nodes in the network, r is the number of nodes in the motif ( r=3 ), ${TF}_{n}$ is the number of TFs in the network, and ${TF}_{m}$ is the number of TFs required for each motif type ( ${TF}_{m}=3$ for 3-feedback loops, and ${TF}_{m}=2$ for feedforward and complex feedforward loops). We exclusively scaled the values of each property vector across networks to the range between 0 and 1. Then, we clustered networks and properties using Ward’s method. Furthermore, we used the pairwise Pearson correlation method to analyze the network property profiles and clustered the networks according to the Euclidean distance using Ward’s method.

### Reconstruction of networks’ hierarchy

We classified each network edge (a, b) as “descendent” if $k_{a}^{out}>k_{b}^{out}$ (where $k_{n}^{out}$ is the out-connectivity of node *n*); otherwise, it was classified as “ascendant.” Then, we removed all the “ascendant” edges from the network. This step removed the feedback present in the network, transforming it into a directed acyclic graph. Then, we applied a modified topological sorting algorithm that returned the list of layers composing the hierarchy, where each node in a layer can only regulate nodes in lower layers. As the number of “ascendant” edges is low (<5% on average), our strategy maintains the global structure of the network to reveal the hierarchy. Additionally, no nodes are removed in the process, and “ascendant” edges can be added back to the hierarchy to indicate the feedback among layers.

## Results and discussion

Matrices of 1,647 unique genes, including matrices of 380 TFs genes, were analyzed using the RSAT matrix-clustering method for the MM *R etli* CFN42 TRN (Castro-Mondragon et al., 2017). One thousand two hundred twelve clustered genes with at least 1 TF included in the cluster, called “clustered-TF-genes,” were selected to construct the TRN. Five hundred twenty gene clusters were formed (Supplementary Table 1 a); from this, 1,146 genes, including 380 TFs and 12 sigma factors, were integrated into the network (Supplementary Table 3 a and c column AH). For the TRN *R. etli* CFN42 bacteroid profile, matrices of 1,241 genes, including the TFs, were analyzed with the matrix-clustering method. Only 939 clustered-TF genes were grouped into 467 clusters (Supplementary Table 1 b). From this, 884 unique genes containing 364 TFs and 12 sigma factors were integrated into the network (Supplementary Table 4 a and c column AG) (see Material and Methods). Notably, selected clusters for these networks were composed of at least two different genes, as clusters are frequently formed with matrices of the same gene, and each gene may have one to five matrices deduced with the RSAT footprinting discovery algorithm (Hertz et al., 1990; Defrance et al., 2008).

For the *S. meliloti* 1021 free-living network, matrix-clustering analysis of matrices from 681 genes, including 260 TFs, resulted in 296 clusters (Supplementary Table 2 a). For the *S. meliloti* 1021 bacteroid TRN, matrices of 372 genes, including 210 TFs, were analyzed with the matrix-clustering method, which resulted in the formation of 181 clusters (Supplementary Table 2 b); these data were used to construct the networks as mentioned above. For free-living clustered-TF *S. meliloti* 1021 TRN, 643 genes, including 259 TFs and eight sigma factor, were integrated (Supplementary Table 5 a and c column AG). Regarding the clustered-TF bacteroid TRN of *S. meliloti* 1021, 357 genes, including 210 TFs and six sigma factors, were integrated (Supplementary Table 6 a and c column AL). In this report, more significant genes and sigma factors were integrated into the *R. etli* CFN42 networks than those in our last report, see above, similar to the *S. meliloti* 1021 networks (Taboada-Castro et al., 2022).

### Strict level of data distribution in networks

One of the challenges in bioinformatic studies is the elimination of false-positive data; one strategy to diminish false-positive data in inferred networks was by lowering TF gene–target relationships in the *p*-value range of e-04 because a low homology between the motif and the sequence of the matrix in this threshold is accepted. It is desirable that TF gene–target relationships be in the range of *p*-values of e-05 and e-06 and lower, which are with medium and strict homology data, respectively. The MM *R. etli* CFN42 network has only 0.41% of data in the *p*-value range e-04 from 9,017 TF gene–targets deduced (Supplementary Table 3 b). Whereas for the bacteroid network, 0.04% of 6,913 TF gene–targets were considered (Supplementary Table 4 b). Moreover, for the free-living *S. meliloti* 1021 network, 0.33% of data in the *p*-value range of e-04 of a network with 4,209 TF gene–targets (Supplementary Table 5 b) and 0.87% of 2,538 TF gene–targets were deduced in the bacteroid network (Supplementary Table 6 b). All these networks are composed of approximately 60% of TF gene–target relationships in the *p*-value of e-06, except for the bacteroid *S. meliloti* network with 56%. These data showed that all these networks were inferred with a quality TF gene–target relationship distribution. However, the occurrence by a chance of motif without a role in the transcription activation is not discarded (Minch et al., 2015).

### Hierarchy of networks and the most regulated genes

The hierarchy of the networks is related to the giant component of the networks; a component is a group of nodes connected by at least one path; networks may have more than one component, and the giant component is the largest component of the network, where the global TFs play a determinant role, that is, in the *E coli* K12 network 511145_v2022_sRDB22_eStrong, the sigma factor *rpoD* has regulatory connections with 1,387 genes; the immediate inferior is *crp* with 547 targets, *rpoS* with 286, fis with 239, IHF with 233, *hns* with 200, *fnr* with 196, *arcA* with 180, *rpoH* with 142, *rpoE* with 115, and the rest of the regulators of transcription with less than 100 targets, and the more regulated genes were *csgG* with 17, *csgF* 17, *csgD* 17, *gadX* 15, *gadA* 12, *flhD* 12, *flhC* 12, and *yhiD* 11 times regulated (Escorcia-Rodríguez et al., 2020). A hierarchy analysis with a network constructed with the breadth-first layout of Cytoscape was carried out. This algorithm defines the gene RHE_RS17050 as a node root, visiting all the next nodes directly from the node root, generating the first level, and so on, until there are no more nodes; this represents the total number of connections (Akram and Dagdeviren, 2013). For the MM *R. etli* CFN42 network hierarchy, RHE_RS17050 potentially has 49 connections, with most of them as regulators, RHE_RS00185 with 47, RHE_RS20580, which is the RNA polymerase factor sigma-32, with 44, and RHE_RS05945 with 43 connections, identified as a LuxR family transcriptional regulator (Supplementary Table 3 c). The five most regulated genes were RHE_RS09360, RHE_RS09355, RHE_RS24105, RHE_RS00340, and RHE_RS01550; those are between 30 and 25 times more regulated (Supplementary Table 3 d). For the bacteroid *R. etli* CFN42 network, the hierarchy based on the node root RHE_RS06405, for the RHE_RS06405, RHE_RS00185, RHE_RS00415, RHE_RS17835, RHE_RS02315, and RHE_RS20580 TFs, is between 45 and 28 times more connected (Supplementary Table 4 c). The five most regulated genes were RHE_RS00340, RHE_RS06680, RHE_RS22960, RHE_RS17050, and RHE_RS16205 in the range of 28–19 times (Supplementary Table 4 d).

In the free-living *S. meliloti* 1021 network, based on the node root SMc02523, the most connected TFs were SMc02523, SMc02470, SMc01593, SM_b21641, SMc02172, SMc00785, and SMc03873, identified as sigma-32 *rpoH2*, with ranges from 28 to 19 connections (Supplementary Table 5 c), and the five most regulated genes were SM_b20129, SM_b21080, SMc04134, SMc02172, and SMa2215 genes, with gradually diminishing times in the range of 23 to 19 (Supplementary Table 5 d). Meanwhile, in the bacteroid *S. meliloti* 1021 network, based on the node root SMc02172, the most connected TF, SMc02172, SMa1207 identified as a Crp family transcriptional regulator, SMc02553, SMc00241, SM_b21222, SMa0498, and SMc04134 are connected between 20 and 15 times (Supplementary Table 6 c). Moreover, the five most regulated genes were SMc02172, SMa2215, SMa0830, the nitrogenase nifE gene for the synthesis of the molybdenum cofactor of the nitrogenase enzymatic complex, SMa1505, and SMc04348, which are regulated in the range of 16 to 12 times (Supplementary Table 6 d).

A comparison of the *E. coli* K12 genomic condition-independent network and partial networks of a dependent condition from these symbiotic species showed they have one giant component (see below). However, the hierarchy is different between them; *E. coli* K12 has a hierarchy by the *rpoD* sigma factor, whereas for these symbiotic species, the hierarchy diminishes gradually. Consequently, the properties of the networks were different (see below). The times a gene is regulated between the two *R. etli* CFN42 networks is almost double that in the *E. coli* K12 network. It is because of the inferred nature of the deduced *R. etli* CFN42 and *S. meliloti* 1021 networks. We have constructed a 93 TF–matrices *E. coli* K12 network with genomic matrix-scan data deposited in the RegulonDB (Tierrafría et al., 2022). This deduced network showed that the most regulated gene is *pdhR*, with 14 times; in contrast, it was only five times in the biological network, and the leuO gene was 13 times, whereas in the biological network, it was seven times (data not shown). From these data, it is estimated that deduced networks have more targets than biological networks.

### TFs specifically encountered in MM or free-living and symbiotic stages

Subtractions of the TFs from MM *R. etli* CFN42 and bacteroid *R. etli* CFN42 networks showed 41 TFs encountered only in MM, and between them, the most represented family of TFs is the LysR family with seven members: RHE_RS01475, RHE_RS04065, RHE_RS23465, RHE_RS24350, RHE_RS24725, RHE_RS26465, and RHE_RS30790. They are followed by five members of the LacI family: RHE_RS27560, RHE_RS28220, RHE_RS00380, RHE_RS27925, and RHE_RS18520; three members of the XRE family: RHE_RS26290, RHE_RS13450, and RHE_RS21900; two AraC family members: RHE_RS07315 and RHE_RS28855; and two ArsR family members: RHE_RS15800 and RHE_RS27600. This highlights the presence of the DNA-directed RNA polymerase sigma-70 factor RHE_RS25560 (Supplementary Table 3 e). Meanwhile, in the bacteroid *R. etli* CFN42 network, 25 TFs were found only in bacteroid, and the most represented family was LysR with seven members: RHE_RS03595, RHE_RS04655, RHE_RS04705, RHE_RS05415, RHE_RS05750, RHE_RS28160, and RHE_RS28900 for diverse functions such as metabolism quorum sensing, motility, and virulence (Maddocks and Oyston, 2008). Interestingly, four members of the TetR/AcrR family, RHE_RS02400, RHE_RS17430, RHE_RS23110, and RHE_RS23915, are related to antibiotics production, osmotic stress, efflux pumps, and multidrug resistance (Deng et al., 2013). The ArsR family RHE_RS05020 gene, the RHE_RS25170 gene, is a member of the Lrp/AsnC family (Thaw et al., 2006). Two genes of the MarR family, RHE_RS01410 and RHE_RS06025, are related to response to chemical signals, degradation of organic compounds, and control of virulence gene expression (Deochand et al., 2017). The AraC family member RHE_RS13370 is related to controlling the expression of virulence genes of pathogenic bacteria and for a sense of environmental chemicals (Yang et al., 2011). The LacI family member RHE_RS12065 is probably involved in regulating carbohydrate metabolism genes (Ravcheev et al., 2014). In response to xenobiotic elements, XRE family gene RHE_RS29835 was identified, and it is probably involved in drug resistance (Miotto et al., 2022). MraZ RHE_RS14605 gene is also probably involved in cell division (Supplementary Table 4 e) (Fisunov et al., 2016). There were 16 TFs more in MM than in bacteroid conditions, and it is because the bacteria are in an exponential growth phase, whereas the bacteroids are in a maximal peak of nitrogen fixation activity. Growth and nitrogen fixation are highly energy-demanding stages, and each had seven LysR family TFs. This shows that bacteroids have a higher content of TFs to contend against the stresses related to the environment than MM.

Free-living *S. meliloti* 1021 network registered exclusively 82 TFs and three sigma factors. There were nine lysR family TFs: SMa0303, SMa0557, SMa1602, SMa1720, SMa1736, SMa1979, SMa1987, SMa2027, and SMa2287; four LacI family TFs: SM_b20667, SM_b21187, SM_b21272, and SMa0078; four ArsR family TFs: SM_b20758, SM_b21008, SM_b21576, and SM_b21601; two AraC family TFs: SM_b21419 and SM_b21559; two TetR family TFs: SMa1726 and SMa2387; one ROK family member: SMa 2004; one MerR family gene: SMA1705; one MUCR family TF SMA1705; and 53 TFs of unknown function. The sigma-32 factors were SMc00646 and SMc03873. The *rpoN* sigma54 factor SMc01139 is also used for nitrogen metabolism (Supplementary Table 5 e).

For the Bacteroid *S. meliloti* 1021 network, 34 TFs and two sigma factors were found only in the symbiotic stage. They included five LysR family TFs: SMa0750, SMa0985, SMa1632, SMa1966, and SM_b21291; five GntR family TFs: SMa0062, SMa0065, SMa0267, SMa0789, and SMa1505; two AraC family TFs: SM_b21385 and SM_b21649; one *irr* for iron response: SMc00329; and one MerR family member, the *hmrR2* SM_b21579 gene. The *ada* TF SMc01728 was for DNA methylation damage response (Landini and Volkert, 1995). The *betI* gene SMc00095 is involved in synthesizing the osmoprotectant glycine betaine (Subhadra et al., 2020). Twelve TFs were of unknown inferred function. Two two-component response regulators: SMa1688 and SM_b20869, as well as two sigma factors, the *rpo* RNA polymerase sigma factor and the *rpoE4* sigma-E, also called sigma-24, were identified with the locus tag SMc04051. Accordingly, it was shown that *rpoE4* was expressed under microaerobic conditions (Supplementary Table 6 f) (Martínez-Salazar et al., 2009).

There were 48 TFs more in the free-living stage than in the bacteroid stage of *S. meliloti* 1021, which suggests a greater regulatory complexity for growth than for nitrogen fixation in *S. meliloti* 1021. This difference was also observed in MM and bacteroid networks of *R. etli* CFN42, as seen above. Notably, the number of TFs between *R. etli* CFN42 and *S. meliloti* 1021 was different. The temperate climate symbiosis of *S. meliloti* induces indeterminate nodules on the roots of the alfalfa plant. In contrast, the tropical symbiosis of the *R. etli* CFN42 induces determinate nodules in the bean plant; in both types of nodules, the metabolism and the differentiation of the bacterium inside the nodules are different, which were reviewed by Liu et al. (2018) and Green et al. (2019). Experiments of co-occurrence are ongoing to determine if these TFs are exclusive for each metabolic condition.

### Inferred regulons

Regulons are groups of genes sharing a TF motif, representing a network’s basic structure. In the MM *R. etli* CFN42 network, 380 regulons were formed. Analyzing each regulon allows us to identify the genes that a TF potentially regulates only in a positive sense. One question is whether the regulon is related to one specific function; from a global view, the request is not easy. A detailed revision of the RHE_RS00185 regulon showed that it is mainly composed of two genes for the production of energy, five genes for the synthesis and metabolism of amino acids, six genes for carbohydrate metabolism, 18 TFs, and four for membrane metabolism, among others. Regulons are ordered by the COG letter, as shown in the “COG group” column; in the column “number of the gene,” each of the genes is enumerated; this lets us know when the genes are neighbors, such as the case for the genes 35 and 36, 58 and 59, 291 and 293, 2,428 and 2,429, 3,117 and 3,118, 3,949 and 3,950, 4,476 and 4,477, and 5,293 and 5,294. Frequently, TFs regulate the neighbor gene or operons; then, it is expected to find TFs in these neighbor genes; Genes 35, 59, 2,429, 3,118, 3,950, and 5,294 are TF genes, and they likely are regulating their neighbor genes (Supplementary Table 3 f, column CD) (Williams and Bowles, 2004; Esch and Merkl, 2020). These observations strengthen the idea that these data are not by chance and that the presence of the motif in the TF and the target–genes has a functional role; however, other possibilities are not discarded.

The bacteroid *R. etli* CFN42 network contains 364 regulons; it is smaller than the MM network. The first regulon listed, RHE_RS00185, is a common TF found in MM and bacteroid networks. This regulon comprises two genes for carbohydrate transport and metabolism, two for coenzyme transport and metabolism, two for cell wall/membrane envelope biogenesis, five for signal transduction mechanisms, and five for LacI family TFs. A search of neighbors based on the numeration genes showed 892 and 893, 2,301 and 2,302, 2,726 and 2,727, and 4,722 and 4,723. From these, the TF genes were 893, 2,301, 2,727, and 4,723, strongly suggesting that they regulate their accompanying genes (neighbors) or operons (Supplementary Table 4 f, column CB). It is frequent to find paralogous TF genes in the regulons, as is the case of five LacI family TF genes in the regulon RHE_RS00185, showing conservation of the motif in the family, suggesting that this motif is not by chance (Supplementary Table 4 f, column CA).

The free-living *S. meliloti* 1021 network contains 259 regulons; the SMa0063 regulon is composed of six TFs annotated only as transcriptional regulators: one gene for translational, ribosomal structure and biogenesis of ribosomal proteins, one gene for cell wall/membrane/envelope biogenesis, and two genes for hypothetical proteins. Only genes 767 and 768 were neighbors; gene 767 is a 50S ribosomal protein L6, and gene 768 is a GntR family TF gene (Supplementary Table 5 f, column BZ).

The bacteroid *S. meliloti* 1021 network contains 210 regulons, and it is smaller than the free-living *S. meliloti* 1021 network; given the size of this network, the presence of genes with the same COG group is lesser than that in the free-living network. These regulons contain a significant number of TFs; taking advantage of this, TFs of the same family per regulon were searched. The SMA0062 regulon contains two GntR family TFs, as is the case for the SMa0065 regulon. The regulon SMa0097 contains two LysR family TFs. The regulon SMa0179, with three genes, contains the genes 5,142 and 5,143, where gene 5,142 is a sugar ABC transporter substrate-binding protein and gene 5,143 is the TF (Supplementary Table 6 g, column CS). The SMa0222 regulon contains two GntR family regulators. The SMa0372 regulon contains the neighbor genes 5,002 and 5,003, where gene 5,002 is an iron ABC transporter substrate-binding protein and gene 5,003 is of the LacI family TF. The SMa0520 regulon contains two LysR family TFs. The SMa0748 regulon contains two GntR family TFs and the 4,457 and 4,458 neighbor genes, where 4,457 gene is a TF and 4,458 gene is a hypothetical protein (Supplementary Table 6 g, column CS). The presence of paralog TF genes grouped in the same regulon, which consequently also share a motif, strongly suggests these regulons are not by chance, and it is highly probable that they are functionally related.

### Neighbor genes expressed in networks

It was shown above that some regulons frequently are with contiguous genes, and one of the genes is a TF. Therefore, an analysis of the neighborhood taking the distance of three genes between them was performed (see Material and Methods) (Taboada-Castro et al., 2020). The MM *R. etli* CFN42 network showed 271 groups with two or more expressed contiguous genes; neighbor genes are denoted with the same number starting from number 0 (Supplementary Table 3 g, column CT), that is, the 0 neighbor genes are gene 5,941 RHE_RS30900, gene 5,940 RHE_RS3314,0 and gene 5,939 RHE_RS30980, with RHE_RS33140 being the nitrogen fixation TF protein. The size of the content of the grouped neighbor genes varies; for example, group 67 contains ten genes located in both the (+) and (−) strings, which contain the *phaR* TF RHE_RS20540, a CarD family TF RHE_RS20575, the factor sigma-32 RHE_RS20580, the MarR family TF RHE_RS20595, and the GntR family TF RHE_RS20625 (Supplementary Table 3 g, column CT). The bacteroid *R. etli* CFN42 network showed 208 groups of neighbor genes. The first group of neighbor genes 0 contains five genes; because this group is not annotated a TF, they are numerated from 5,879 to 5,983, the NoiL RHE_RS31100 gene, the aquaporin RHE_RS31105 gene, the phasin RHE_RS31110 gene, the adenine phosphoribosyltransferase RHE_RS31115 gene, and the radical SAM/SPASM domain-containing RHE_RS31120 gene. One of the representative numerous groups of neighbor genes is the 124; it contains eleven genes, from 2,542 to 2,562 enumerated genes: the DNA-binding response regulator RHE_RS13180, the TF RHE_RS13190, the TetR/AcrR family TF RHE_RS13215, the O-methyltransferase RHE_RS13220, the TF RHE_RS13235, the SDR family NAD(P)-dependent oxidoreductase RHE_RS13240, the hypothetical gene RHE_RS13255, the VOC family RHE_RS13265 gene, the 4-hydroxy-tetrahydrodipicolinate reductase RHE_RS13275, the AraC family TF RHE_RS13295, and the dehydrogenase RHE_RS13300 (Supplementary Table 4 g, column CR).

The free-living *S. meliloti* 1021 network contains 126 grouped neighbor genes. The first neighbor group enumerated with 0 was constituted of the carbon–phosphorus lyase SM_b20759, the ArsR family TF SM_b20758, and the propionyl-CoA carboxylase subunit beta SM_b20755. One of the numerous groups of neighbor genes was the 111 group numerated from 473 to 487; it contains ABC transporter permease SMc02160, glucose-6-phosphate isomerase SMc02163, dihydroorotase SMc02166, ABC transporter permease SMc02170, TF SMc02172, hypothetical gene SMc02174, and TF SMc02175 (Supplementary Table 5 g, column CO). Moreover, the bacteroid *S. meliloti* 1021 network contains 54 neighbor groups, and it is the smallest network in this report. The first group enumerated with 0 contains three genes, the 6,214 to 6,217 enumerated genes, composed of LacI family TF SM_b21650, AraC family TF SM_b21649, and alpha-galactoside ABC transporter substrate-binding precursor SM_b21647. Group one comprises the *paaX* for the ArsR family TF SMb_21641 and the *paaA* phenylacetate-CoA oxygenase subunit SM_b21640. Group two contains the two genes, the LacI family TF SM_b20674 and the sugar-uptake ABC transporter ATP-binding SM_b20673. The most numerous groups contain four to five genes; for example, the group of neighbor genes 10 contains the ferredoxin reductase *mocF* gene SM_b20820, the ferredoxin *mocE* gene SM_b20819, the hydrocarbon oxygenase *mocD* gene SM_b20818, and the LacI family TF SM_b20817 (Supplementary Table 6 h, column DG).

A global view of neighbor genes showed that there is an expression of groups of genes that are contiguous and frequently contain TF or TFs probably involved in the positive transcriptional regulation of their contiguous genes. In line with that, some regulons contain neighbor genes, and consequently, they share a motif (see above). As a gene may be regulated for more than one TF, valuable information for experimentalists to design experiments on transcriptional regulation is to consider the neighborhood of the genes of interest. However, other genes in the regulons may or may not be neighbors because for the construction of the networks, the TF and the target gene with the lowest *p*-value were prioritized, and because regulation of genes at a distance was shown (see Material and Methods) (Williams and Bowles, 2004; Pannier et al., 2017; Esch and Merkl, 2020).

### Distribution of regulon’s genes in modules of WGCNA

Because the *S. meliloti* 1021 TRNs were from one transcriptome analysis (Barnett et al., 2004), we wanted to know if these free-living and bacteroid profiles correspond to modules, and where the exclusively TF genes per condition were grouped. A Weighted Gene Co-expression network analysis (WGCNA) was carried out with 40 transcriptomes (Langfelder and Horvath, 2008; Galán-Vásquez and Perez-Rueda, 2019). Transcriptomes were fished in the COLOMBOS database using a bacteroid profile gene from this study (Moretto et al., 2016). WGCNA identifies modules using unsupervised hierarchical clustering, without the use of *a priori* defined gene sets. Modules are defined as clusters of densely interconnected genes by comparing the correlation of expression levels of the genes. Genes from regulons from the free-living *S. meliloti* 1021 TRN were searched in the modules. The module turquoise grouped 37.12% of genes, brown 30.0%, blue 19.15%, and pink 9.56%. Meanwhile, the exclusive free-living TFs were distributed in the turquoise module at 30.59%, blue 25.88, brown 25.88%, and pink 16.47% (Supplementary Table 5 h column CX). In contrast, genes from the regulons of the *S. meliloti* 1021 bacteroid network were grouped in the pink module at 30.82%, blue at 28.26%, turquoise at 21.8%, brown at 12.38%, and black at 4.71%. Meanwhile, the bacteroid-exclusive TFs were in turquoise module at 30.58%, pink at 27.78%, blue at 22.22%, black at 11.11%, and brown at 8.33% (Supplementary Table 6 i column DN). These data showed that the pink module contains the greater number of bacteroid regulon’s genes, whereas the turquoise model grouped the greatest number of regulon’s genes for free-living conditions. Although the turquoise module contains, in both profiles, a higher number of genes, it was expected because in *R. etli* CFN42 TRNs, there are a large number of genes shared in these conditions (Taboada-Castro et al., 2022). Concerning the exclusive TFs from free-living and bacteroid conditions, some were grouped in the turquoise module. Notice that the pink and blue modules contain a second and third lower number of TFs in the bacteroid. Whereas in the free-living condition, the pink module contained a low number of exclusive free-living condition TFs (Supplementary Table 5 h column CX; Supplementary Table 6 i column DM). This shows that the pink module is grouping the symbiotic nitrogen fixation genes from the bacteroid network. Of 58 *nod* and *fix* genes for nodulation and nitrogen fixation, 35 genes were found in the pink module, 13 in blue, five in turquoise, three in brown, one in magenta, and one in the tan module (data not shown). These data showed that for the *S. meliloti* 1021 regulons of the TRNs, the turquoise and the pink modules from the WGCNA grouped most of the genes from free-living and bacteroid conditions, respectively. This established a relationship between regulon and co-expression of genes in modules from the WGCNA. In addition, the data from one experiment are a reliable sample to infer a TRN.

### Properties of the networks

#### Disposition of hierarchic TFs in a network

**FIGURE 1:**
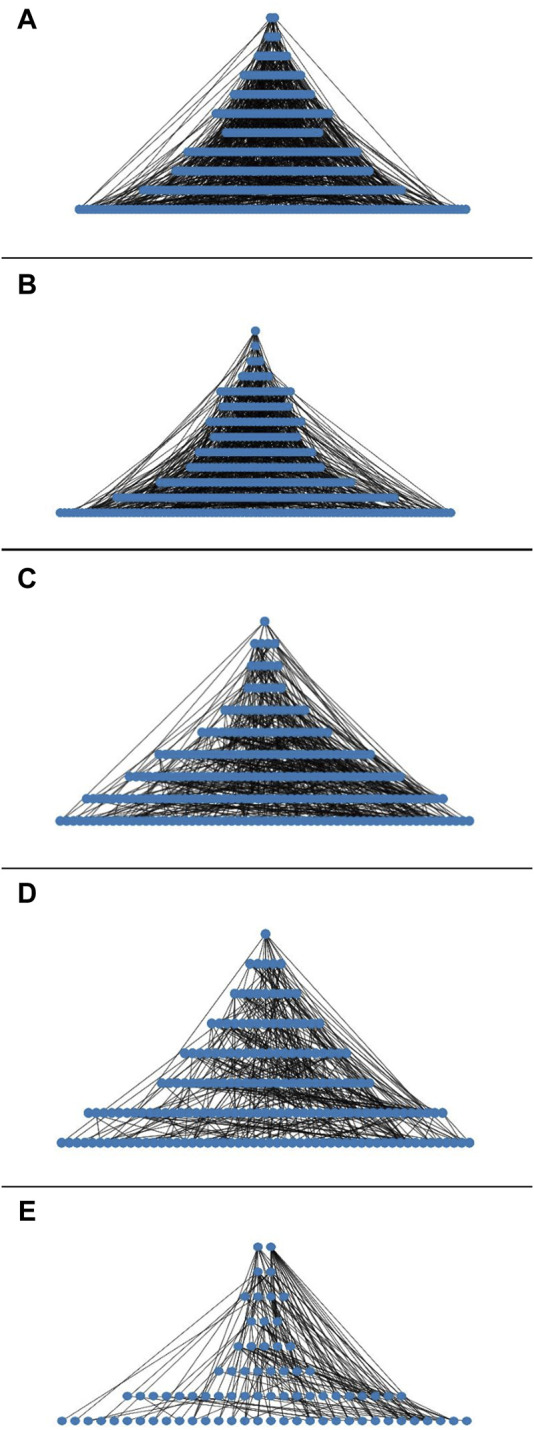
**(A)** Hierarchy of the transcriptional regulatory networks of: **(A)** minimal medium *R. etli* CFN42, **(B)** bacteroid 18 days *R. etli* CFN42, **(C)** free-living *S. meliloti* 1021, **(D)** bacteroid *S. meliloti* 1021, and **(E)** *E. coli* K12 93 TFs.

Taking advantage of the network constructed for the analysis of the properties and to compare with our last report, we search for the hierarchy looking for a pine-tree-like structure (see Material and Methods) (Taboada-Castro et al., 2022). TF–TF relationships showed the basic hierarchy structure of the networks; they showed a top-down pyramidal structure (See Figures 1A–E). In MM, *R. etli* CFN42 network showed 11 levels compared to clustered-TF-MM, which showed seven levels (Taboada-Castro et al., 2022). At level_1, the RHE_RS15800 ArsR family transcriptional regulator and the RHE_RS17050 DNA-binding response regulator were identified. At level_2, the RHE_RS05945 LuxR family transcriptional regulator, the RHE_RS05495 ArsR family transcriptional regulator, and the RHE_RS06405 transcriptional regulators were found. The first sigma factor, RHE_RS17090 RNA polymerase sigma factor *RpoH*, was found at level_5. The next sigma factor, RHE_RS20580 sigma-32, was found at level_6; RHE_RS26680 was located at level_7, sigma factor RHE_RS15090 rpoD at level_9, and RHE_RS26695 sigma-70 at level_10. At level_11, five sigma factors, RHE_RS32370, RHE_RS05265 sigma-70, RHE_RS28625, RHE_RS02045 *rpoN* sigma-54, and the RHE_RS03775 sigma factor, were located. Compared to the *E. coli* global network, where the sigma factor *rpoD* sigma-70 is at the top (Freyre-González et al., 2008), in the *Bacillus subtilis* global network, the *sigA* is at the top (Freyre-González et al., 2012). In this MM *R. etli* CFN42-dependent condition network, the first sigma factor appeared until level_5, showing that the basic structure of these networks was different (Figure 1A; Supplementary Table 7 a). Accordingly, a condition-dependent network of *E. coli* K12 showed *rpoH* in a down position compared to that in its global network (Resendis-Antonio et al., 2005; Gutierrez-Ríos et al., 2007). These data suggest that the physiological conditions shape the network.

In addition, the bacteroid *R. etli* CFN42 network contained 13 levels (Figure 1B), whereas for the last report, in the clustered-TF bacteroid network, we showed four levels (Taboada-Castro et al., 2022). At level_1, the RHE_RS06405 transcriptional regulator was identified. At level_2, the RHE_RS17835 ArsR family transcriptional regulator was located. At level_3, the RHE_RS25040 transcriptional regulator, the RHE_RS20580 RNA polymerase factor sigma-32, and the RHE_RS23635 Lrp/AsnC family transcriptional regulator were located. Additionally, at level_8, sigma RHE_RS26680 was found. At level_10, RHE_RS15090 *rpoD* was identified. At level_11, sigma factor RHE_RS32370 and RHE_RS26695 sigma-70 were located. At level_12, sigma RHE_RS28625 and RHE_RS17090 *rpoH* were located. Moreover, at level_13, six sigma factors, RHE_RS20110 *rpoE*, RHE_RS02045 *rpoN* sigma-54, sigma RHE_RS15675, RHE_RS05265 sigma-70, RHE_RS23540, and RHE_RS03775, were identified (Figure 1B; Supplementary Table 7 b, column S). *R. etli* contains 23 sigma factors, and it has shown the presence of canonical extracytoplasmic sigma factors groups G1 and G2; EcfG1 and EcfG2 are canonical and non-canonical, respectively (Martínez-Salazar et al., 2009). Neither of these two groups of sigma factors appears to play a significant role during symbiotic nitrogen fixation (Jans et al., 2013). These data confirmed that the sigma factors do not play a hierarchal top role for the MM and bacteroid networks.

The free-living *S. meliloti* 1021 network constructed with transcriptome data showed eight levels (Figure 1C) (Barnett et al., 2004). For level_1, TF SMc02172 was identified. At level_2, rirA iron-responsive TF SMc00785 and TFs SM_b21598, SMc03816, and SMc01593 were identified. At level_3, *paaX* ArsR family TF SM_b21641, TFs SMc02425 and SMc04134, LysR family TF SMa0498, and TetR family TF SMa5030 were identified. At level_4, the first sigma factor-32 *rpoH2* as well as 5 TFs, LysR family SM_b20582 and SMc00163, LacI family SM_b21187, SMc01945, and SMc01762 were located. Meanwhile, at level_5, the plasmid located *gstR*-like TF SM_b20004, MucR family TF SMa0748, and tetR family TF SM_b21208 were identified altogether with 10 TFs; SMc00241, SMc03820, SMa0748, SM_b21021, SMc02504, SMc02523, SM_b21115, SMc00679, SMc03046, and SMc02984 were found. For level_6, three sigma factors, the first sigma-32 rpoH1, rpoE5, SM_b21484, and the RNA polymerase sigma factor SMc04203, together with 17 TF, were found. At level_7, together with 32 TFs, *tacA* sigma-54-dependent TF SMc04011 was found. At level_8, 42 TF was found, including the RNA polymerase sigma factor SM_b20531. In addition, for level_10, three sigmas, *sigA*, *rpoD* SMc01563, *nifA*, SMa0815, and the *rpoN* sigma-54 factor, together with 75 TFs, were identified (Figure 1C; Supplementary Table 7 c, column AF). In contrast to the free-living network, the bacteroid *S. meliloti* 1021 network was less complex, and it contained eight levels (Figure 1D). At level_1, the unknown function TF SMc02172 was found. At level_2, four TFs, including two plasmid-borne TFs, Crp family TF SMa1207, and the SMa0498 LysR TF, were found, accompanied by TFs SMc04134, SMc03122, and SMc00278. At level_3, the MucR family TF plasmid-encoded SMa0748, the plasmid-borne lysR SM_b20582, the *chvI* chromosomal encoded SMc02560, and the TetR family TF plasmid-encoded SM_b21208 in addition to six TFs, SM_b20162, SM_b21222, SMc02470, SMc04163, SMc02323, and SMc02560, were detected, and in level_4, the first sigma *fecl* chromosomal-encoded SMc04203, the *cbbR* TFR, *betI* SMc00095, *irr* chromosomal-encoded SMc00239 iron response regulator, and the *paaX* ArsR family TF were found, in addition to the nine TFs: SM_b20457, SM_b20901, SMc00241, SMa200, SM_b21598, SMc02523, SM_b21115, SMa0267, and SMa5030. The second sigma factor in this network appeared at level_7; the plasmid-encoded SM_b20531 was identified, in addition to the *nifA* SMa0815, and the sigma-54-dependent TF *tacA* SMc04011, in addition to *gstR* SM_b20004, *deoR* SM_b21299, *gbpR* SMb20896, and *exoX* SM_b20947, and the response regulator *cpaE1* were detected, in addition to 40 TFs. In level_8, three sigma factors, the plasmid-encoded *rpo* SM_b20592, *rpoE5* (sigma-24) plasmid-borne SM_b21484, and the *rpoE4* (sigma-24) chromosomal-encoded SMc04051, were detected. In addition, *rirA* SMc00785, *ctrA* SMc00654, *actR* SMc02584, *algR* SMc03060, *agpT* SM_b21649, and *syrM* SMa0849 were detected, in addition to 54 TFs (Figure 1D; Supplementary Table 7 d, column AS).

In addition, a hierarchy analysis of an independent condition *E. coli* K12 partial network was included, which was constructed with deduced matrices of 93 TFs taken from RegulonDB (Tierrafría et al., 2022). The 93 TFs *E coli* K12 network showed eight levels, and their network properties will be discussed in the next sections (Figure 1E). Altogether, the deduced networks showed a top-down pyramidal structure showing the hierarchy among TFs.

On the role of sigma factors in Rhizobium, it was shown that the 11 extracytoplasmic sigma factors of the *S. meliloti* 1021 strain were dispensable for free-living growth and nitrogen fixation activity during symbiosis with the *Medicago truncatula* alfalfa plant, suggesting that they control accessory functions (Lang et al., 2018). Then, it was shown that the role of the sigma factors is different between species.

### Analysis of the networks

#### Similarity of networks

**FIGURE 2:**
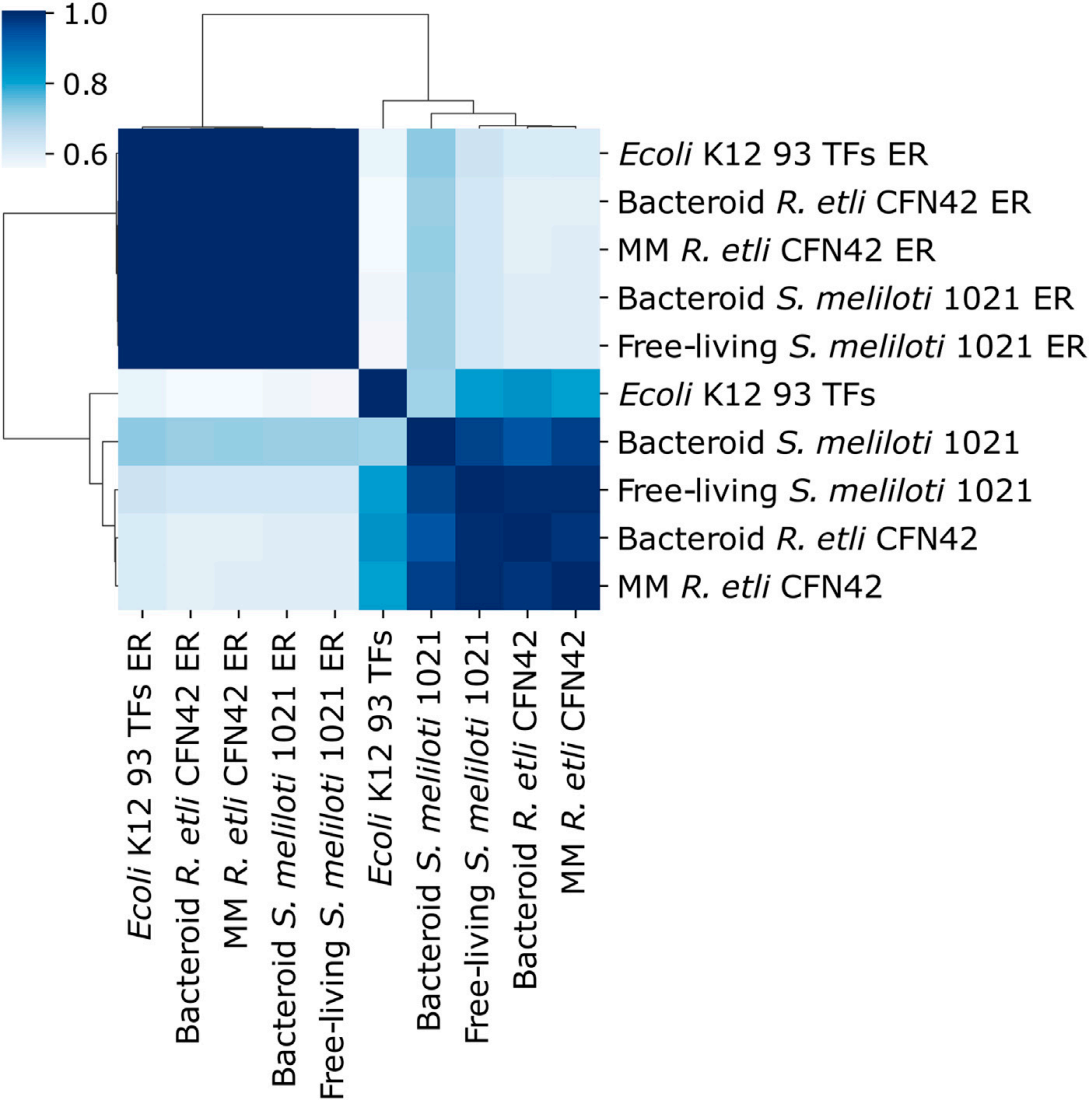
Symmetry analysis between the deduced transcriptional regulatory networks of minimal medium and bacteroid from *R. etli* CFN42, free-living and bacteroid from *S. meliloti* 1021, *E. coli* K12 93 TFs, and their respective Erdös–Rényi random networks (ER_avg).

The deduced MM *R etli* CFN42, bacteroid *R. etli* CFN42, free-living *S. meliloti* 1021, bacteroid *S. meliloti* 1021, and 93 TFs *E. coli* K12 networks were compared with their respective random networks (ER) constructed according the Erdös–Rényi model (Figure 2) (see Material and Methods). Deduced networks segregate from their respective ER networks, meaning that deduced networks are not random. Bacteroid *S. meliloti* 1021 networks had a low similarity with their respective ER networks. Moreover, *R. etli* CFN42 and *S. meliloti* 1021 networks were more similar to the 93 TFs *E. coli* K12 network.

#### Properties of the networks

**FIGURE 3:**
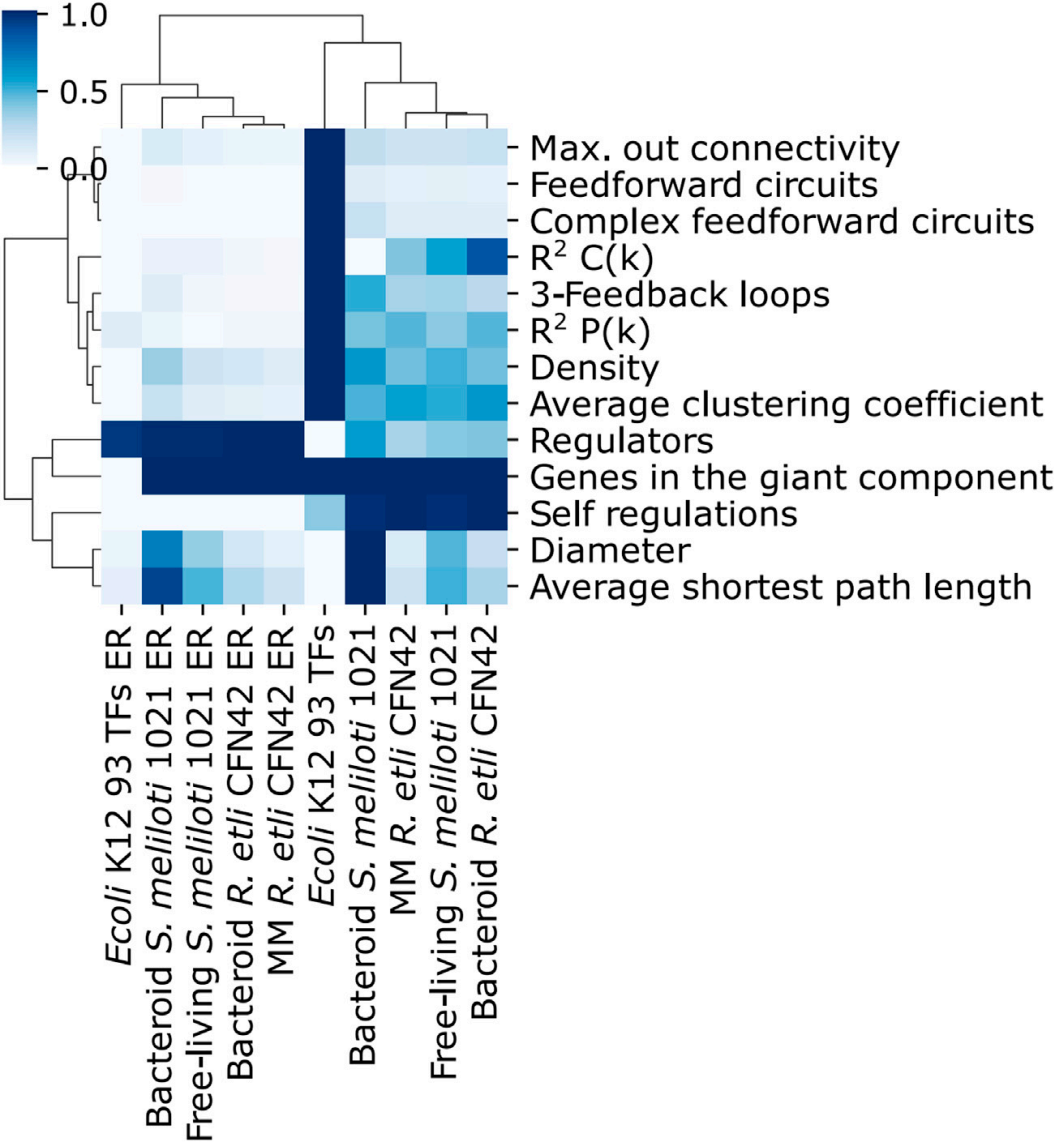
Comparison of properties of the transcriptional regulatory networks of minimal medium and bacteroid from *R. etli* CFN42, free-living and bacteroid from *S. meliloti* 1021, and *E. coli* K12 93 TFs.

Maximal out connectivity, feed-forward circuits, complex feed-forward circuits, the distribution of the average clustering coefficient R^2^ C(K), 3-feedback loops, the distribution of the connectivity of nodes R^2^ P(K), density, average clustering coefficient, and self-regulations were different between the deduced and ER networks (Figure 3). For the deduced networks, 93 TFs *E. coli* K12 network had greater values than the *R. etli* CFN42 and *S. meliloti* 1021 deduced networks; however, the number of genes in the giant component was similar. Self-regulations were more remarkable for the *R. etli* CFN42 and *S. meliloti* 1021 networks than for the 93 TFs *E. coli* K12 networks because these deduced networks were constructed only with TFs whose matrices were able to recognize a motif in their own upstream promotor. These differences among the control 93 TFs *E. coli* K12 network, *R. etli* CFN42, and *S. meliloti* 1021 networks are because of their structure and not because of their incompleteness because the 93 TFs *E. coli* network is also an incomplete independent condition network. In addition to that, they are different species; in the same species, the dependent and independent conditions of the networks are also possibly different.

#### Proportion of TFs and structural genes

**FIGURE 4:**
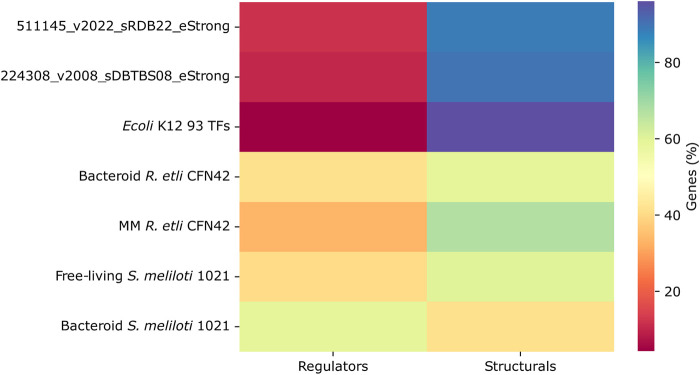
Proportion of transcriptional regulators and structural genes in the transcriptional regulatory networks of minimal medium and bacteroid from *R. etli* CFN42, free-living and bacteroid from *S. meliloti* 1021, and *E. coli* K12 93 TFs.

The deduced *R. etli* CFN42 and S. *meliloti* 1021 networks as compared with the 93 TFs *E. coli* K12, 511145_v2022_sRDB22_eStrong *E. coli* K12 complete network, and 22438_v2008_sDBTBS08_Strong *B. subtilis* network contained a significative lower number of structural genes. In contrast, the *R. etli* and *S*. *meliloti* networks contained a greater number of TFs (Figure 4). This probably favors major connectivity and density for the 93 TFs *E. coli* K12 network (see Figure 3).

#### Kmax of the TFs

**FIGURE 5:**
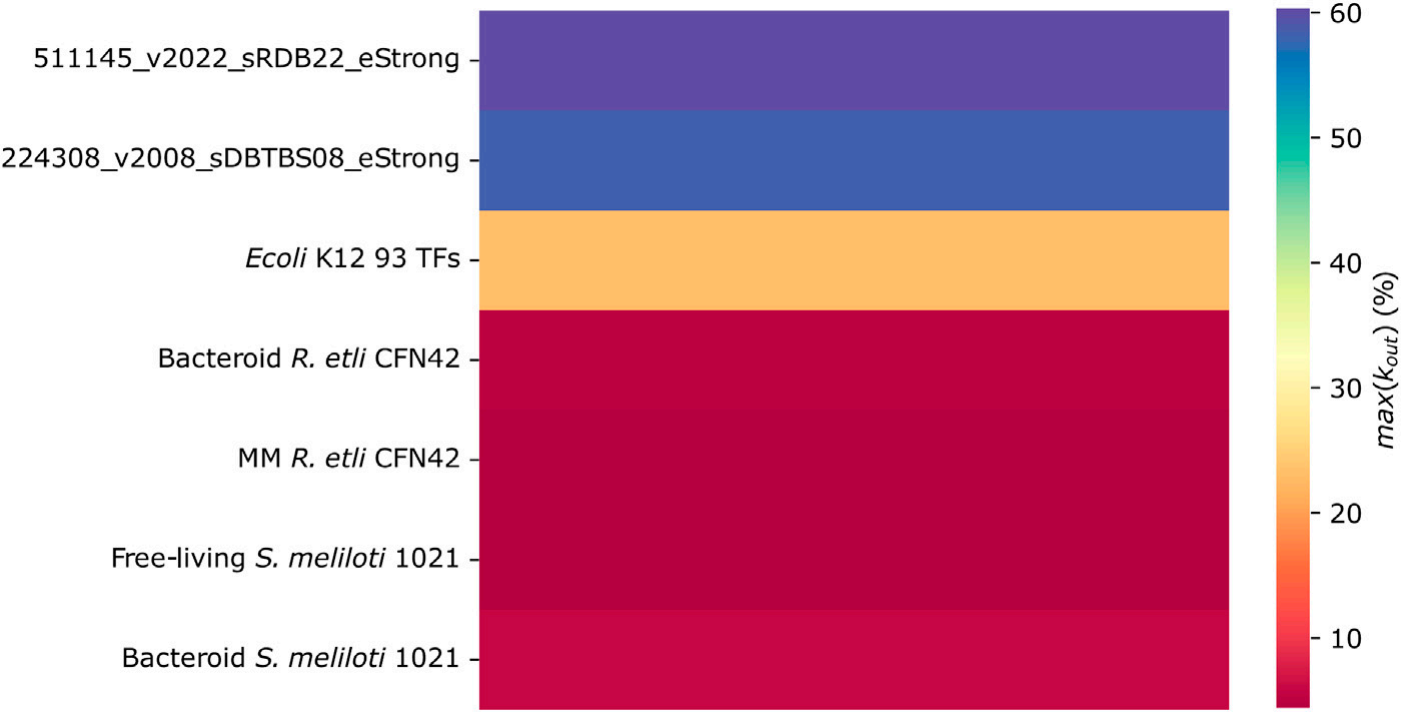
Number of links between genes (Kout) in the transcriptional regulatory networks of minimal medium and bacteroid from *R. etli* CFN42, free-living and bacteroid from *S. meliloti* 1021, and *E. coli* K12 93 TFs.

The number of genes regulated by a TF is defined by their Kout, and the Kmax parameter defines the maximum value of Kout. As was shown above, probably, the low content of structural genes is diminishing the Kmax of the *R. etli* CFN42 and *S. meliloti* 1021 networks as compared with the most complete *E. coli* K12 and *B. subtilis* networks and the predicted 93 TFs *E. coli* K12 network (Figure 5). In other words, the connectivity of the main hub in *R. etli* and *S. meliloti* 1021 networks is low compared to that of the main hub in *E. coli* K12 and *B. subtilis* networks.

#### Shortcuts in the networks

**FIGURE 6:**
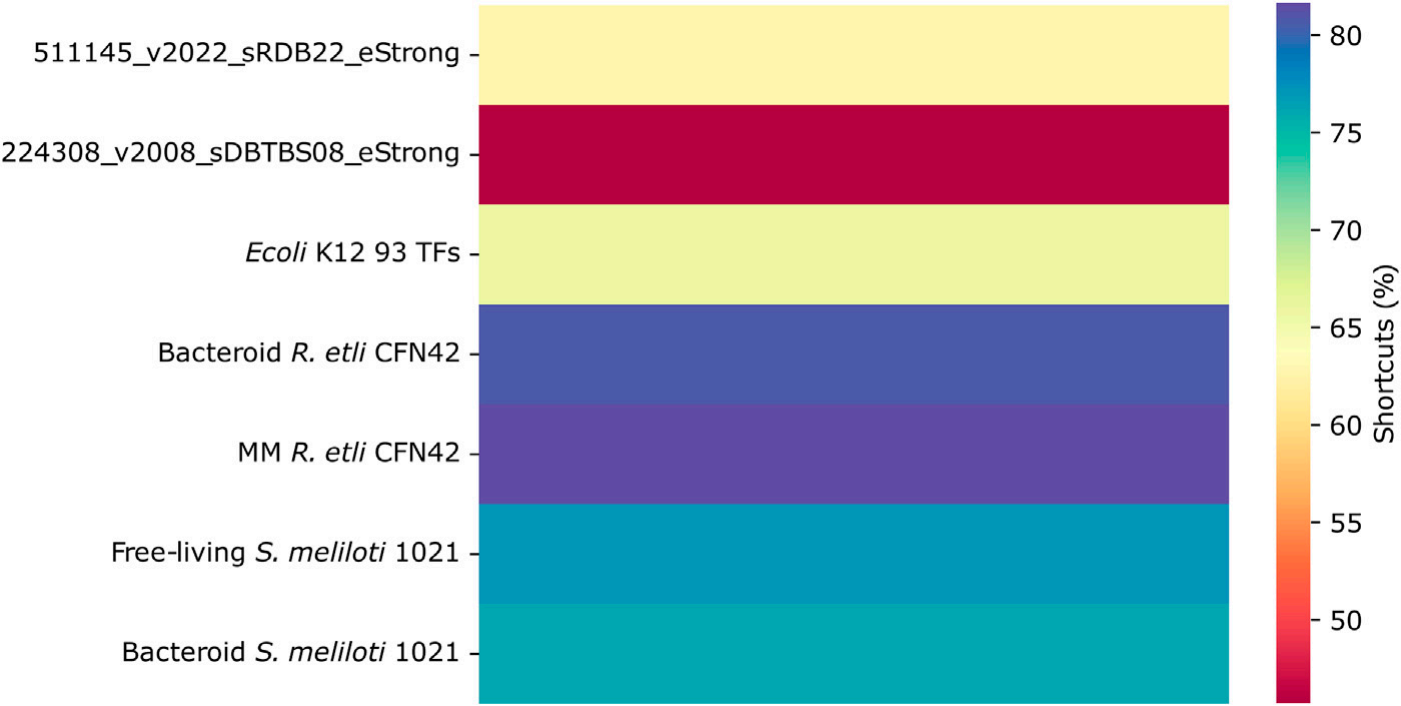
Shortcuts in the transcriptional regulatory networks of minimal medium and bacteroid from *R. etli* CFN42, free-living and bacteroid from *S. meliloti* 1021, and *E. coli* K12 93 TFs.

The navigability of a network is increased in small-world networks due to the presence of shortcuts and hubs. The percentage of shortcuts is more significant for the *R. etli* and *S. meliloti* 1021 networks than for the deduced and most complete *E. coli* K12 and *B. subtilis* networks (Figure 6). It is not clear whether these network structures pose any functional advantages.

#### Kolmogorov–Smirnov distance

**FIGURE 7:**
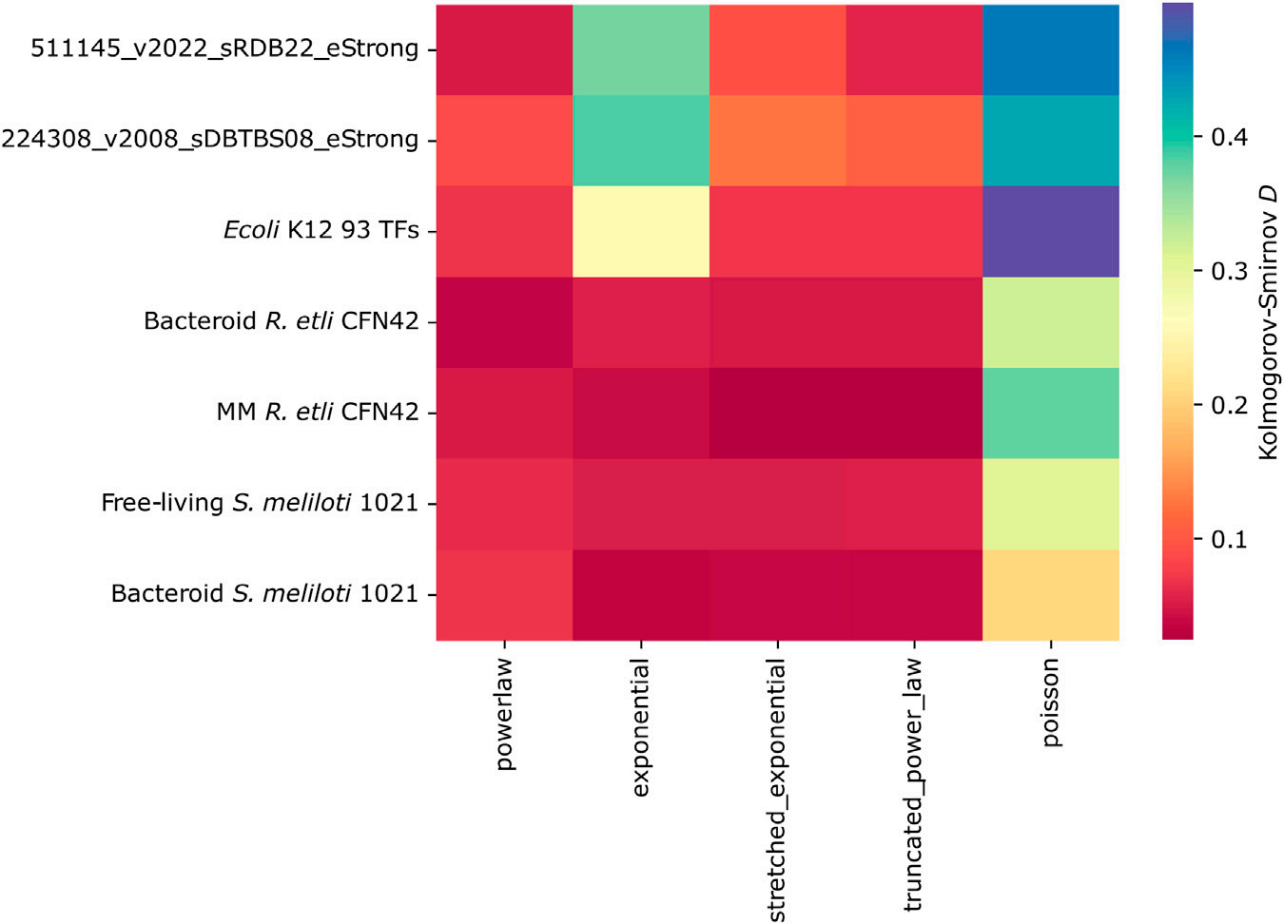
Kolmogorov–Smirnov distance of the transcriptional regulatory networks of minimal medium and bacteroid from *R. etli* CFN42, free-living and bacteroid from *S. meliloti* 1021, and *E. coli* K12 93 TFs.

We assess the goodness of fit of each network connectivity distribution to alternative long-tail probability distributions. The *E. coli* K12, *B. subtilis*, and 93 TFs *E. coli* K12 networks showed a preference for power-law or truncated power-law, clearly showing that exponential is not a *bona fide* model for the connectivity distributions of these networks. On the contrary, *R. etli* and *S. meliloti* 1021 networks cannot exclude this possibility as the differences among the exponential and the alternative distributions are tiny. Bacteroid *S. meliloti* 1021 is the only network showing a slight preference for power-law (Figure 7). However, the low presence of hubs strongly suggests that the deduced *R. etli* and *S. meliloti* 1021 networks are not scale-free.

#### The presence of replicons in the deduced networks

*A priori* that these deduced *R. etli* CFN42 and *S. meliloti* 1021 networks were experimentally tested, they showed a gradually diminishing hierarchy, and these dependent condition networks are not comparable to the independent condition hierarchy network of *E. coli*. Condition-dependent networks are a subgroup of a global network. It has shown that species with similar lifestyles conserve their regulatory motif networks, even with high genetic variability between them, which suggests evolutionary pressure. Therefore, they conserved similar logical responses or strategies (Madan Babu et al., 2006; Galardini et al., 2015). Notably, *E. coli* and the symbiotic species *R. etli* and *S. meliloti* 1021 have crucial lifestyle differences. Rhizobia lives in the rhizospheric environment and as a symbiont in the plant cell. *R*. *etli* CFN42 contains six plasmids, and *S*. *meliloti* contains a chromid and a megaplasmid (Landeta et al., 2011; Galardini et al., 2015). It was shown that these extrachromosomal entities are indispensable for growth, meaning that they coded for essential functions of the bacterial cell; for example, the plasmid p42e of *R. etli* CFN42 code for the synthesis of cobalamin, cardiolipin, cytochrome *o*, NAD, and thiamine, as well as the genes for the septum formation; in addition to that, there are homologous replicons in other symbiotic species (Landeta et al., 2011; Martínez-Absalón et al., 2022). The *S. meliloti* chromid and megaplasmid contain genes for metabolism and symbiosis, respectively (Galardini et al., 2015). Therefore, probably special evolutionary forces are on the symbiotic species; for example, it was suggested that adaptive evolution, genome innovation, and reconstruction of the regulatory networks conferred the nodulation and the nitrogen fixation ability of these species (Liu et al., 2023). Then, replicon-specific wiring of the regulatory network in the *S. meliloti* strain was suggested, and this inference may be occurring for all the symbiotic species (Galardini et al., 2015). In line with this, isoenzymes of 34 enzymes were detected in MM and bacteroid protein profiles of *R. etli* CFN42, suggesting independent transcriptional regulation (Taboada-Castro et al., 2022). Accordingly, TFs for only MM and symbiosis were indicated in this study (see above).

#### Information to design experiments on transcriptional regulation

In addition to the Supplementary Material included in this study, the RhizoBindingSites v1.0 database contains all the information on TFs and their potential targets from nine rhizobia species (Taboada-Castro et al., 2020) (http://rhizobindingsites.ccg.unam.mx/). These data were divided into low, medium, and high stringency. By introducing the name of the TF in the motif information window, the output data of the potential targets of the TF at the genomic level are provided, similarly to as was shown in the networks (Supplementary Tables 3–6 a) (see user’s guide of RhizoBindingSites). In this table, a column “matrix-ID” containing the names of the matrices of the TF and the nucleotide sequence of the sites is shown. The matrix is provided by clicking on the name of the matrix, and a new window appears with the matrix in the transfact format with the “Motif Logo” and “Motif Map” applications that display the logo of the motif and the conservation of this motif in the promoter regions of the ortholog genes of the TF, respectively. In addition, as was shown in the Material and Methods section of this study for the construction of a network, to construct hypothetical regulons from profile data, the co-expressed TF genes with structural genes of a profile are used by copy-pasting the TFs in the left box of the application “prediction of regulatory networks” and the structural genes with the TFs in the right box. Then, the “auto” option is selected and run (submitted “RhizoBindingSites v2.0 is a bioinformatic database of DNA motifs potentially involved in transcriptional regulation deduced from their genomic sites”) (Taboada-Castro et al., 2022). In the RhizoBindingSites v1.0 database, to experimentally search the motif, the nucleotide sequence of the site, the matrix representing the site, the logo of the matrix, the conservation of the motif in the ortholog genes, and the potential target genes of a TF displayed in a graph (hypothetical regulon) are provided.

## Conclusion

In the era of high-throughput technologies, prote**omic** and transcript**omic**, “omic” data from bacterial culture cells and symbiosis of *R. etli* CFN42 and *S. meliloti* 1021 were used to construct four transcriptional regulatory networks. A new method based on the selection of genes clustered by the matrix-clustering analysis was used to build these networks, and now, a more significant number of genes were integrated into the *R. etli* CFN42 networks than in our last report (Taboada-Castro et al., 2022).

The *R. etli* CFN42 and *S. meliloti* 1021 deduced networks were constructed with motifs conserved in both the TF and the gene–target. A highly strict TF gene–target distribution of data per network was shown. Computational construction of these networks shows valuable information on the hierarchy of the TFs for each network, including the times a gene is potentially regulated, the presence of specific TFs per network, the regulons per network, neighbor genes containing TFs in the regulons or in the networks, and the construction of TRNs with one transcriptome data of *S. meliloti* 1021. A more significant number of TFs related to the environment were found in the bacteroid than in the MM networks of *R. etli* CFN42.

It was shown that the deduced networks were segregated from the random Erdös–Rényi networks. They were more similar to the biological *E. coli* and *B. subtilis* networks. Deduced *R. etli* CFN42 and *S. meliloti* 1021 networks are not scale-free because of a low average clustering coefficient, low maximal connectivity, and the absence of *bona fide* hubs, probably by a fractionated origin of the network, which contains genes from the chromosome, chromids, or plasmids. In contrast to the biological networks, for deduced networks, the physiological condition may be re-organizing the circuitry of the network.

The use of bioinformatic methods becomes fundamental to deducing conserved motifs involved in transcriptional regulation, as well as constructing transcriptional regulatory networks. Using the inferred data of transcriptional regulation to design better experiments will accelerate the knowledge of, for example, regulons, which are the bases for a transcriptional regulatory network.

## Data Availability

The datasets presented in this study can be found in online repositories. The names of the repository/repositories and accession number(s) can be found in the article/Supplementary Material.
